# Inhibition of activator protein 1 attenuates neuroinflammation and brain injury after experimental intracerebral hemorrhage

**DOI:** 10.1111/cns.13206

**Published:** 2019-08-08

**Authors:** Chang‐Juan Wei, Yu‐Lin Li, Zi‐Long Zhu, Dong‐Mei Jia, Mo‐Li Fan, Ting Li, Xue‐Jiao Wang, Zhi‐Guo Li, Hong‐Shan Ma

**Affiliations:** ^1^ Tianjin Neurological Institute Tianjin Medical University General Hospital Tianjin China; ^2^ Center for Neuroinflammation, Beijing Tiantan Hospital Capital Medical University Beijing China; ^3^ Department of Neurology Tianjin Huanhu Hospital Tianjin China; ^4^ Center for Neurological Diseases The Third People's Hospital of Datong Datong China

**Keywords:** activator protein 1, brain injury, intracerebral hemorrhage, microglia, neuroinflammation

## Abstract

**Aims:**

Intracerebral hemorrhage (ICH) is a devastating type of stroke without specific treatment. Activator protein 1 (AP‐1), as a gene regulator, initiates cytokine expression in response to environmental stimuli. In this study, we investigated the relationship between AP‐1 and neuroinflammation‐associated brain injury triggered by ICH.

**Methods:**

Intracerebral hemorrhage mice were developed by autologous blood or collagenase infusion. We measured the dynamics of AP‐1 in mouse brain tissues during neuroinflammation formation after ICH. The effects of the AP‐1 inhibitor SR11302 on brain injury and neuroinflammation as well as the underlying mechanisms were investigated in vivo and in vitro.

**Results:**

AP‐1 was significantly upregulated in mouse brain tissue as early as 6 hours after ICH, accompanied by elevations in proinflammatory factors, including interleukin (IL)‐6, IL‐1β, and tumor necrosis factor (TNF)‐α. Inhibition of AP‐1 using SR11302 reduced neurodeficits and brain edema at day 3 after ICH. SR11302 ablated microglial IL‐6 and TNF‐α production and brain‐infiltrating leukocytes in ICH mice. In addition, SR11302 treatment diminished thrombin‐induced production of IL‐6 and TNF‐α in cultured microglia.

**Conclusions:**

Inhibition of AP‐1 curbs neuroinflammation and reduces brain injury following ICH.

## 1 INTRODUCTION

Intracerebral hemorrhage (ICH) is a severe disease with high morbidity and mortality and lacks effective management.^1^, ^2^, ^3^ Following the onset of ICH, blood components enter the brain and activate resident microglia.^4^, ^5^, ^6^ Concomitant with subsequent leukocyte infiltration, activated microglia augment the local production of proinflammatory cytokines, which amplifies blood–brain barrier (BBB) disruption and accelerates the expansion of perihematomal edema (PHE), resulting in more severe and durable brain injury.^7^, ^8^, ^9^ Although emerging evidence has implicated inflammation as a major component of PHE in the acceleration of brain injury following hemorrhage,^7^, ^10^, ^11^, ^12^, ^13^ the action of brain‐intrinsic factors in injury‐induced neuroinflammation remains poorly defined in ICH.^14^

Activator protein 1 (AP‐1) is generally a heterodimer composed of proteins of the Jun, Fos and activating transcription factor protein (ATF) families.^15^, ^16^ As a transcription factor, AP‐1 switches on targeted gene expression and upregulates cytokine expression, contributing to tissue inflammation in diseases such as rheumatoid arthritis, psoriasis, and psoriatic arthritis.^17^, ^18^ In acute brain injury such as ICH, however, little is known about the role of AP‐1 in neuroinflammation formation. Using a mouse model of ICH induced by injection of autologous blood or collagenase, we determined the expression and contribution of AP‐1 to neuroinflammation and hemorrhagic brain injury.

## 2 MATERIALS AND METHODS

### 2.1 Mice

C57BL/6 male mice 10‐12 weeks old were used in this study. All mice were housed in the animal facilities at Beijing Tiantan Hospital and Tianjin Neurological Institute. All experiments were approved by the guide for the Care and Use of Laboratory Animals from the Committee on the Ethics of Animal Experiments of Beijing Tiantan Hospital and Tianjin Neurological Institute. Mice were randomly assigned to groups and given anesthesia during surgeries.

### 2.2 ICH model

#### 2.2.1 Autologous blood injection model

An autologous blood injection model was induced in mice as described previously.^19^, ^20^, ^21^ Briefly, after mice were anesthetized with an intraperitoneal injection of 5% chloral hydrate at a dose of 8 mL/kg body weight, 30 μL nonheparinized autologous blood was withdrawn from the angular vein and infused into a 50 μL microliter syringe with a 26 G needle (Hamilton). For autologous blood injection, we drilled a 1 mm diameter hole (2.3 mm right lateral to midline and 0.5 mm anterior to bregma) in the skull of the mice. Five microliters of blood was injected into the brain parenchyma at 3 mm depth from the hole of the skull, and the remaining 25 μL of blood was injected at 3.7 mm depth at 1 μL/min. After 30 μL blood was injected, the needle was left in place for 20 minutes prior to withdrawal.

#### 2.2.2 Collagenase injection model

For the collagenase injection model, a hole similar to that in the above autologous blood injection model was drilled, and 0.0375 U bacterial collagenase IV‐S in saline (Sigma) was injected into the brain parenchyma at 3.7 mm depth with a rate of 0.5 μL/min. Following injection, the needle was left in place for 5 minutes before it was withdrawn. During the surgeries, mouse body temperature was maintained by a 37°C heat lamp. After the needle was withdrawn, the hole in the mouse skull was sealed, and the skin incision was sutured. The mice were then transferred to cages and kept in an animal room with free access to food and water.

### 2.3 SR11302 administration

SR11302 (Tocris) was administered via brain parenchymal injection 1 hour before ICH induction. Briefly, SR11302 stock solution was dissolved in dimethyl sulfoxide (DMSO) at a concentration of 100 mmol/L. Mice were anesthetized through intraperitoneal injection of 5% chloral hydrate at a dose of 8 mL/kg body weight. A total of 1 μL 100 mmol/L SR11302 was injected into the brain parenchyma at a rate of 0.5 μL/min at 3.7 mm depth under the drilled hole in the skull at 2.3 mm lateral to midline and 0.5 mm anterior to bregma, whereas 1 μL DMSO was administered to mice of the control group. For in vitro cell culture experiments, a 100× working solution of SR11302 at 1 mmol/L was prepared by adding 1 μL storage solution into 99 μL saline. The dosages of SR11302 for these experiments were chosen in reference to previously published studies.^22^, ^23^

### 2.4 Neurological deficit assessment

Neurological tests were performed blindly by no less than two investigators at 24 hours and 72 hours after ICH. We used the modified neurological severity score (mNSS) and corner‐turn test to evaluate neurodeficits as previously described.^19^, ^24^ The mNSS is scaled from 0 to 15 points, with a point given for each test according to the performance of the mouse and the scoring standard, providing an overall score for each mouse. For the corner‐turn test, the mouse was placed in a corner with a 30° angle, and the turning direction for exiting the corner was recorded. Each mouse performed each trial 10 times with an interval of 30 seconds between trials, and the percentage of right turns was calculated.

### 2.5 Brain water content assessment

To assess brain water content, ICH mice were euthanized and decapitated at 72 hours after ICH. Brain tissues were immediately divided into the left hemisphere, right hemisphere, and cerebellum after isolation from the head. Each of the three parts was then weighed to obtain wet weight and then dried for 24 hours at 100°C to obtain the dry weight. Brain water content was calculated using the following formula: (wet weight − dry weight)/wet weight × 100%.

### 2.6 Immunofluorescence

Immunofluorescence staining was performed as previously described.^19^, ^24^ Paraffin‐embedded brain tissue from ICH and sham mice was cut into 5 μm continuous coronal sections. Sections were permeabilized and incubated with blocking solution consisting of 5% donkey serum and 0.3% Triton‐X 100, followed by incubation with antibodies against Iba1 (Abcam) and c‐Jun (Abcam) at 4°C overnight. After the tissue was washed with PBS solution 3 times, the slices were incubated with fluorochrome‐conjugated antibodies (Invitrogen) at room temperature for 2 hours. Then, the slices were washed with PBS 3 times before being incubated with Fluoroshield Mounting Medium with DAPI (Abcam). Images were taken with a fluorescence microscope (model BX‐61, Olympus).

### 2.7 Flow cytometry

Single‐cell suspensions were prepared from brain tissues and stained with fluorochrome‐conjugated antibodies similar to previous studies.^25^, ^26^ Cells were stained with antibodies purchased from BD Bioscience or eBioscience, Inc. If not otherwise indicated. Antibodies were directly labeled with one of the following fluorescent tags: fluoresce isothiocyanate (FITC), phycoerythrin (PE), PerCP‐Cy5.5, or allophycocyanin (APC), and PE‐Cy7. Antibodies targeting the following mouse antigens were used: CD45 (eBioOMAK‐D), CD3 (145‐2C11), NK1.1 (PK136), CD11b (M1/70), F4/80 (6F12), interleukin (IL)‐1β (B122), IL‐6 (MQ2‐6A3), tumor necrosis factor (TNF)‐α (MP6‐XT22), Ly6G (1A8), glial fibrillary acidic protein (GFAP) (1B4), NeuN (A60) (Sigma‐Aldrich), and isotype IgG. AP‐1 was stained with a c‐Jun primary antibody (Abcam) and a donkey anti‐rabbit 594 secondary antibody (Invitrogen). For intracellular antigen staining, additional cell fixation and permeabilization were performed according to the manufacturer's protocol of the Fixation/Permeabilization Solution Kit (BD Bioscience). All gates were set using FMO controls. A FACS Aria III (BD Bioscience) was used to collect flow cytometer data, and the data were further analyzed with FlowJo software (Version 7.6.1, FlowJo, LLC).

### 2.8 Cell culture

Microglia were isolated from 10‐ to 12‐week‐old C57BL/6 male mice and purified using flow cytometry. The purity of microglia was confirmed by flow cytometry prior to culture (>98%). Briefly, mice were euthanized, and brain tissues were removed and minced with scissors in ice‐cold Dulbecco's Modified Eagle Medium (DMEM) (Invitrogen). Thereafter, minced tissues were digested with 0.25% trypsin (Invitrogen) in a water bath at 37°C for 30 minutes to form a monocular suspension. After the samples were stained with CD11b and CD45, microglia were sorted on a FACS Aria III flow cytometer (BD Bioscience). After centrifugation, 1 × 10^5^ microglia were seeded in a well of a 24‐well cell culture dish with 1 mL microglia culture medium [DMEM, high glucose + 10% (vol/vol) FBS + 1% penicillin/streptomycin] and cultured within an incubator with 5% CO_2_ and an atmospheric O_2_ concentration at 37°C for further assay. Microglia were incubated with thrombin (2 U/mL) or SR11302 (10 μmol/L) for 24 hours prior to testing their cytokine expression.

### 2.9 Real‐time polymerase chain reaction (PCR)

Total RNA was isolated from ICH brain tissues at 6 hours, 12 hours, and 24 hours after ICH surgery. The purity of the total RNA extracted by Trizol was measured by ultraviolet spectrophotometry. cDNA was synthetized from total RNA using TransScript First‐Strand cDNA Synthesis SuperMix (Transgen Biotech). PCR was performed on an Optical 2 Real‐Time PCR Detection System (Bio‐Rad) with the appropriate primers and SYBR green PCR Master Mix (Roche Diagnostics). The primers used to measure gene expression are listed as follows: c‐Jun (5′‐ ACG ACC TTC TAC GAC GAT GC‐ 3′, 5′‐CCA GGT TCA AGG TCA TGC TC‐3′); c‐Fos (5′‐GGG GAC AGC CTT TCC TAC TA‐3′, 5′‐CTG TCA CCG TGG GGA TAA AG‐3′); IL‐6 (5′‐GAG GAT ACC ACT CCC AAC AGA CC‐3′, 5′‐AAG TGC ATC ATC GTT GTT CAT ACA‐3′); IL‐1β (5′‐TGC CAC CTT TTG ACA GTG ATG‐3′, 5′‐TGA TGT GCT GCT GCG AGA TT‐3′); TNF‐α (5′‐AGG CAC TCC CCC AAA AGA TG‐3′, 5′‐TGA GGG TCT GGG CCA TAG AA‐3′) and β‐actin (5′‐CTG TCC CTG TAT GCC TCT G‐3′; 5′‐ATG TCA CGC ACG ATT TCC‐3′). Each sample was performed in duplicate, and gene expression was normalized to β‐actin expression using the 2–ΔΔCt method. The specificity of primers and PCR reagents were routinely determined by melting curves.

### 2.10 Statistics

Data are expressed as the mean ± SEM. Two‐tailed unpaired Student's *t *test and one‐way ANOVA were used to determine significance between two groups or more than two groups, respectively. Values of *P* < .05 were considered significant.

## 3 RESULTS

### 3.1 AP‐1 was upregulated in the brains of ICH mice

**Figure 1:**
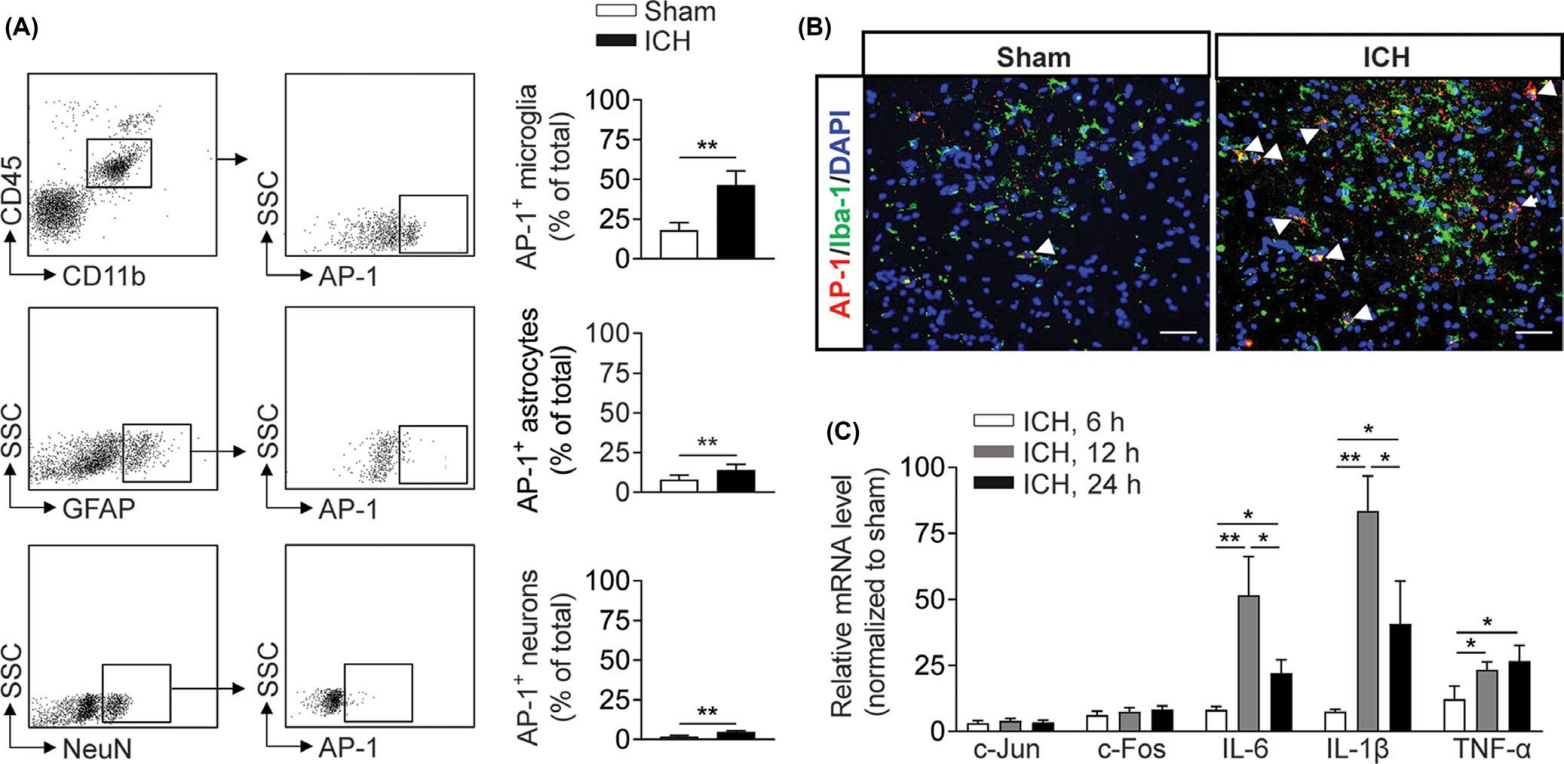
Early upregulation of AP‐1 in the brain of ICH mice. A, Increased expression of AP‐1 in microglia, astrocytes, and neurons from sham and ICH mice. ICH mice were developed by autologous blood infusion. AP‐1 was analyzed by flow cytometry. n = 6 mice per group. B, Immunostaining images showing Iba 1^+^ cells (green) expressing AP‐1 (red) in groups of mouse brain sections harvested at 6 h after ICH or sham procedures. Scale bar: 40 μm. C, Bar graph shows mRNA expression of AP‐1 subunits (c‐Jun and c‐Fos) and inflammatory factors of brain tissues harvested at 6 h, 12 h, and 24 h after ICH or sham procedures in mice. n = 3 mice per group. Mean ± SEM. *P < .05; **P < .01

AP‐1 is an inducible transcription factor receptive to various stresses; therefore, we compared the protein level of AP‐1 in injured brain tissues from autologous blood injection ICH mice vs that in tissues from sham controls. As early as 6 hours post‐ICH, we found a dramatic upregulation of AP‐1 in microglia (Figure 1A). Moreover, AP‐1 was also upregulated in astrocytes and neurons to a lesser extent (Figure 1A). The high expression of AP‐1 in microglia was confirmed by immunostaining of Iba 1^+^ cells in brain sections from ICH mice (Figure 1B). In addition, we measured the mRNA levels of AP‐1 subunits (c‐Jun and c‐Fos) and related inflammatory genes (IL‐6, IL‐1β and TNF‐α) in ICH brain tissues at 6 hours, 12 hours, and 24 hours after ICH onset. Compared with mRNA expression in the sham control group, mRNA expression of AP‐1 subunits including c‐Jun and c‐Fos was elevated at 6 hours after ICH, and mRNA expression of proinflammatory cytokines such as IL‐6, IL‐1β, and TNF‐α was upregulated at 12 hours after ICH (Figure 1C). These findings suggest early upregulation of AP‐1 in microglia after ICH onset.

### 3.2 Inhibition of AP‐1 reduced brain water content and mortality after ICH

**Figure 2:**
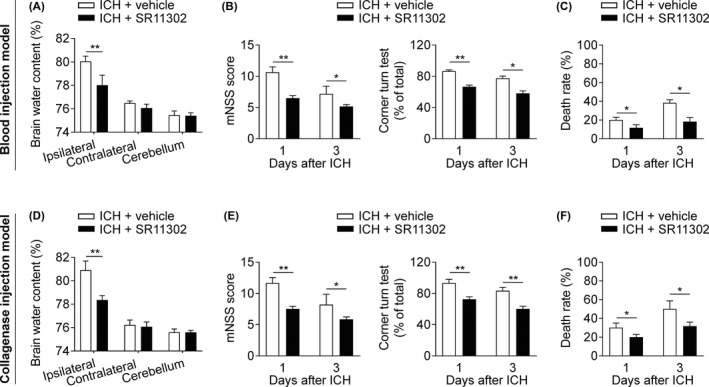
Effects of AP‐1 inhibition on brain edema, neurodeficits, and mortality after ICH. ICH mice were developed by infusion of autologous blood (A‐C) or collagenase (D‐F). SR11302 or vehicle was administered by intracranial injection at 1 h prior to ICH induction. A and D, Brain water content was assessed in ICH groups that received the indicated treatments on day 3 after ICH. n = 6 mice per group. B and E, Neurological outcome in the indicated groups of ICH mice was measured by an assessment of the mNSS and the corner‐turn test. n = 6 mice per group. C and F, Mortality rate in groups of ICH mice who received the indicated treatments. n = 12 mice per group. Mean ± SEM. *P < .05; **P < .01

To understand the potential involvement of AP‐1 in ICH injury, we administered the AP‐1 inhibitor SR11302 to ICH mice. SR11302 treatment reduced the brain water content of the ipsilateral hemisphere in both the blood‐ and collagenase‐induced ICH models (Figure 2A,D). Improved neurological outcomes and lower mortality rates were also found in ICH mice who received SR11302 (Figure 2B,C,E,F). These data indicate that AP‐1 inhibition reduces ICH injury.

### 3.3 Inhibition of AP‐1 reduced neuroinflammation after ICH

**Figure 3:**
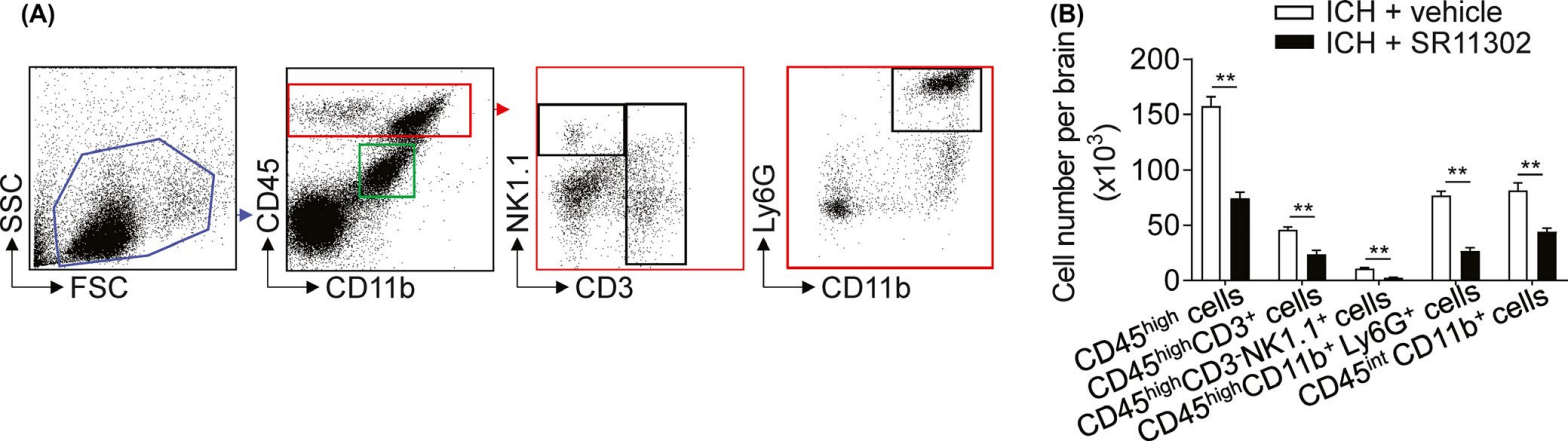
Effects of AP‐1 inhibition on microglia and brain‐infiltrating leukocyte counts after ICH. A, Flow cytometry gating strategy of microglia and brain‐infiltrating leukocytes harvested from the indicated groups of ICH mice. SR11302 or vehicle was given by brain parenchymal injection at 1 h prior to ICH induction. ICH mice were developed by infusion of autologous blood. B, Counts of neutrophils (CD45^high^CD11b^+^Ly6G^+^), T cells (CD45^high^CD3^+^), NK cells (CD45^high^CD3^−^NK1.1^+^), and microglia (CD45^int^CD11b^+^) per brain in the indicated groups of mice at 24 h after ICH. n = 6 mice per group. Mean ± SEM. **P < .01

AP‐1 is highly expressed by microglia, which are thought to be the first responders to ICH and mount local immune responses in the brain; therefore, we assessed the effects of AP‐1 inhibition on leukocyte infiltration and microglia activity in ICH mice. SR11302 treatment reduced brain infiltration of neutrophils, T cells, and natural killer (NK) cells, accompanied by a reduced amount of microglia in the brain at day 3 after ICH (Figure 3A,B).

### 3.4 Inhibition of AP‐1 reduced the production of proinflammatory factors by microglia

**Figure 4:**
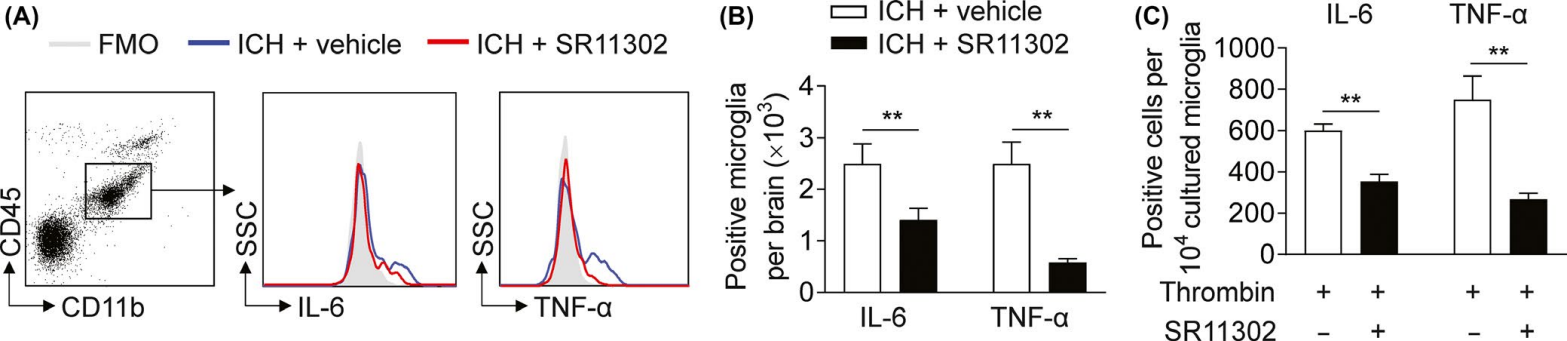
Inhibition of AP‐1 reduced microglial production of IL‐6 and TNF‐α in ICH. A, Microglia expressing IL‐6 and TNF‐α were analyzed by flow cytometry. Microglia were isolated from autologous blood‐induced ICH mice who received SR11302 (red) or vehicle (blue) at 6 h after ICH. B, Quantification of microglia expressing IL‐6 and TNF‐α isolated from the indicated groups of ICH mice. n = 6 mice per group. C, The levels of IL‐6 and TNF‐α in cultured microglia after exposure to thrombin (2 U/mL) + vehicle or thrombin (2 U/mL) + SR11302 (10 μmol/L). Microglia were incubated with thrombin and vehicle or SR11302 at 37°C in an incubator with 5% CO_2_ and an atmospheric O_2_ concentration for 24 h, and their expression of IL‐6 and TNF‐α was analyzed by flow cytometry. n = 3 per group. Mean ± SEM. **P < .01

As a critical transcript factor, AP‐1 is a molecular switch that controls the expression of downstream proinflammatory factors. We therefore investigated the effects of AP‐1 inhibition on the production of proinflammatory factors by microglia after ICH. At 6 hours after ICH, SR11302 treatment ablated the expression of IL‐6 and TNF‐α in microglia (Figure 4A,B). To test whether AP‐1 inhibition directly affects microglia to inhibit their expression of proinflammatory factors, we used an in vitro thrombin model. After exposure to thrombin, cultured microglia displayed upregulation of IL‐6 and TNF‐α. SR11302 treatment diminished the upregulation of IL‐6 and TNF‐α in microglia (Figure 4C). These data indicate that AP‐1 increases the production of proinflammatory factors in microglia in response to ICH stimuli.

## 4 DISCUSSION

This study provides the first evidence that AP‐1 mediates neuroinflammation‐associated brain injury in ICH. Intracerebral hemorrhage rapidly elicits inflammatory responses within the brain that are characterized by the early production of proinflammatory cytokines such as IL‐6 and TNF‐α by neuroglia, which exacerbates BBB disruption and brain edema.^27^, ^28^, ^29^ As presently reported, ICH induced an upregulation of AP‐1 predominantly in microglia within 6 hours after onset. The upregulation of AP‐1 was accompanied by an increase in IL‐6 and TNF‐α, major cytokines known to be transcription ally controlled by AP‐1. Notably, pharmacological inhibition of AP‐1 led to reduced counts of microglia and infiltrating leukocytes, together with attenuated microglial production of IL‐6 and TNF‐α. Collectively, our results suggest a previously unrecognized role of AP‐1 in neuroinflammation and brain injury after ICH.

The inflammatory responses to ICH are characterized by a swift activation of microglia localized in the perihematomal area.^12^, ^30^ Microglia have multiple capabilities that can critically impact hemorrhagic brain injury, including the production of proinflammatory cytokines.^6^, ^31^ Recent studies have demonstrated that activation of microglia contributes to brain injury after ICH^30^, ^32^, ^33^, ^34^ and that removal of microglia attenuates hemorrhagic brain injury.^19^ In line with these findings, ICH triggered a predominant expression of the transcriptor AP‐1 in microglia in conjunction with upregulation of IL‐6 and TNF‐α, well‐known proinflammatory cytokines that amplify local inflammation, further supporting a detrimental role of microglia in acute ICH injury. In addition, although the hematoma components (erythrocytes, heme, thrombin, and complements, etc)^35^, ^36^, ^37^ are known to activate microglia, the precise molecular mechanisms that mediate microglial activity after ICH remain elusive. The identification of the role of AP‐1 provides a better understanding of the intrinsic machinery that acts as a determinant of the inflammatory cascades after ICH.

To understand the possible mechanisms through which inhibition of AP‐1 might provide protection, we measured the immune responses within the brain after ICH. Inhibition of AP‐1 led to diminished numbers of microglia and infiltrating leukocytes along with a corresponding decrease in IL‐6 and TNF‐α expression. In support of this finding, the AP‐1 inhibitor directly mitigated the thrombin‐induced production of IL‐6 and TNF‐α in cultured microglia, indicating that reduced leukocyte infiltration into the brain might result from the AP‐1 inhibition‐induced restriction of microglial responses. However, other myeloid cell types in addition to microglia and astrocytes, including monocytes and macrophages, may also express AP‐1 after ICH, albeit to a lesser extent. Therefore, the benefit of AP‐1 inhibition in ICH may not entirely stem from its effects on microglia. Nevertheless, the precise operating mechanisms underlying AP‐1 inhibition‐induced restriction of brain inflammation after ICH require further investigation.

In conclusion, our results identify AP‐1 as a molecular switch mediating neuroinflammation and brain injury after ICH. The relevance of this finding to human ICH and the value of AP‐1 as a potential treatment target require further investigation.

## CONFLICT OF INTEREST

Authors declare no conflict of interest.
